# Bridging trust gaps: Stakeholder perspectives on AI adoption in the United Kingdom NHS primary care

**DOI:** 10.1177/20552076251386706

**Published:** 2025-11-27

**Authors:** Teresa Sides, Dhouha Kbaier, Tracie Farrell, Aisling Third

**Affiliations:** 1 5488 The Open University, Milton Keynes, UK

**Keywords:** Artificial intelligence, primary health care, trust, health personnel, United Kingdom

## Abstract

**Objectives:**

This study investigates the factors influencing artificial intelligence (AI) acceptance in the United Kingdom (UK) National Health Services (NHS) primary care across different stakeholder levels. It explores key barriers and enablers, focusing on workforce impact, organisational influences and ethical considerations.

**Methods:**

A mixed-methods, between-subjects approach was employed to capture stakeholder perspectives on AI trust and acceptance. Eligible participants were NHS primary care employees working at macro-, meso- and micro-levels. An online survey was distributed via Prolific and included both closed- and open-ended questions. This was followed by semi-structured interviews with a subset of volunteers. Quantitative data were analysed using descriptive statistics and qualitative responses were examined using abductive thematic analysis.

**Results:**

The study gathered 133 responses and conducted nine follow-up interviews. Three key themes were identified: (1) Workforce impact and resistance to change – concerns about job loss and skills gaps; (2) Organisational and cultural barriers – challenges around validation, leadership buy-in and integration; and (3) Trust, ethics and equity – fears about fairness, accountability and the exacerbation of healthcare disparities. Findings revealed that meso-level stakeholders (primary care managers) act as both facilitators and barriers to AI adoption, mediating between macro-level policymakers and micro-level frontline healthcare professionals while navigating organisational constraints and workforce concerns.

**Conclusion:**

The findings underscore the necessity for ethical and trustworthy AI systems, targeted stakeholder engagement, and strategies to ensure equitable and effective AI implementation in primary care. Further research should examine how trust influences AI adoption and equity in care delivery.

## Introduction

The National Health Service (NHS) was established on 5 July 1948 and advocated for a free national healthcare system to promote social equality.^1^ Starfield et al.^2^ described primary care as providing comprehensive person-centred care, tailored to the individual, considering their medical history, lifestyle and social environment. The United Kingdom (UK) government has emphasised the potential of artificial intelligence (AI) to improve the quantity and quality of healthcare services across the NHS.^3^ In recent years, the adoption of AI in healthcare has increased significantly, though it remains predominantly focused on secondary care settings.^4^ In contrast, primary care remains under-researched, with limited empirical evidence to guide implementation strategies.^5^ This is particularly problematic in the UK, where fragmented primary care structures – characterised by decentralised decision-making, varied resource availability and inconsistent digital strategies – complicate AI adoption^.^^6^

In this context, stakeholder trust is pivotal. Trust in AI can vary substantially across professional groups and organisational levels. Macro-level stakeholders (e.g. policymakers) often act as top-down enablers for AI integration^3^ while micro-level stakeholders (e.g. clinicians, nurses and administrative staff) interact directly with AI tools. Meso-level stakeholders (e.g. practice managers) act as intermediaries, shaping day-to-day implementation and operational decisions.^7^ Despite their potential to influence both strategic uptake and frontline use, existing research has largely focused on micro- and macro-level perspectives^8–11^ with limited attention given to meso-level stakeholders, particularly primary care managers, with most existing research focusing instead on technology providers.^12^ Given the growing recognition of their role as implementation intermediaries, further exploration of their perceptions is warranted – alongside continued attention to other key stakeholder levels.

Building on Steerling et al.'s^13^ identification of trust dimensions – including AI system reliability, user trust and regulatory trust – this study examines how these factors manifest across different stakeholder levels in NHS primary care. Notably, high profile failures such as the eating disorder chatbot that provided stakeholders with inappropriate advice have contributed to public and professional mistrust of AI systems.^14^ Ethical concerns, including risks to person-centred care, privacy and accountability, further complicate adoption. Karimian et al.^15^ noted that without sufficient regulatory oversight AI may exacerbate inequities rather than reduce them. Tomaselli et al.^16^ similarly emphasised the importance of ethical alignment with relational person-centred care values. Trust, empathy and ethical alignment are therefore increasingly recognised as essential to fostering responsible AI acceptance in primary care.^8,17^

### Research gap

While existing literature highlights a growing interest in AI adoption within the NHS and the trust related barriers to its acceptance, most studies have predominantly focused on macro-level decision-makers and micro-level practitioners. In contrast, meso-level stakeholders – who often influence implementation, manage staff expectations and interpret policy at the operational level – remain underrepresented in the literature.^7^ This oversight may be due to historical emphasis on either policy design (macro) or end-user interaction (micro), overlooking the intermediary role that managers play in navigating organisational readiness and workforce concerns.

This study investigates how trust-related concerns manifest across different stakeholder levels. Rather than focusing exclusively on one group, this research draws insights from multiple organisational levels – including micro and meso stakeholders – while acknowledging the absence of macro-level interviewees as a limitation.

To address these gaps, this study investigates the following research questions:

1. How do workforce concerns, ethical considerations, and organisational resistance shape trust in AI among NHS primary care stakeholders?
2. Which stakeholder level is perceived to have the most influence over the acceptance of AI for primary care?

## Methods

### Research approach

The study employed a mixed-method, between-subjects design to ensure a comprehensive understanding of stakeholder perspectives on AI in NHS primary care. It was conducted in the UK between February 2023 and September 2023, comprising of two phases: an online survey and follow-up semi-structured interviews. This design enabled within-method triangulation of quantitative and qualitative data.^18^ The target population consisted of primary care employees working at macro-, meso- and micro-levels.

### Survey design

The survey was hosted on Prolific, an established online research platform that provides access to a large pool of participants for academic studies. Eligible participants were NHS primary care stakeholders, aged 18 and over, who had experience in administrative, clinical, or managerial roles. Recruitment quotas were not applied, though the sample included staff across, micro-, meso-, and macro-levels. A pre-screener survey was used to identify the organisational level of the respondents to filter for meso- and macro-level respondents to increase the number of responses adjusting for the high volume of micro-level participants.

The survey instrument was developed by the authors with reference to validated constructs from the Technology Acceptance Model 3 (TAM3)^19^ and the Unified Theory of Acceptance and Use of Technology 2 (UTAUT2).^20^ Permission to adapt elements of these frameworks was obtained from copyright holders, and all survey items were original formulations rather than direct reproductions. These models were selected for their well-established frameworks in technology acceptance, incorporating trust characteristics to address the unique context of primary care. Combining TAM3 and UTAUT2 provided a more comprehensive understanding of stakeholder perceptions of AI, both individually and across various organisational levels. Given the critical role of trust in AI adoption, the study also incorporated trust-related characteristics, such as ‘fairness’, ‘accountability’, ‘transparency’ and ‘ethics’ (FATE), which captures key dimensions previously identified as pivotal in healthcare AI ethics.^21,22^ Integrating these trust dimensions with TAM3 and UTAUT2 allowed for a more nuanced analysis, as trust had emerged as a key determinant of AI acceptance in existing literature.^13^

**Figure 1.**
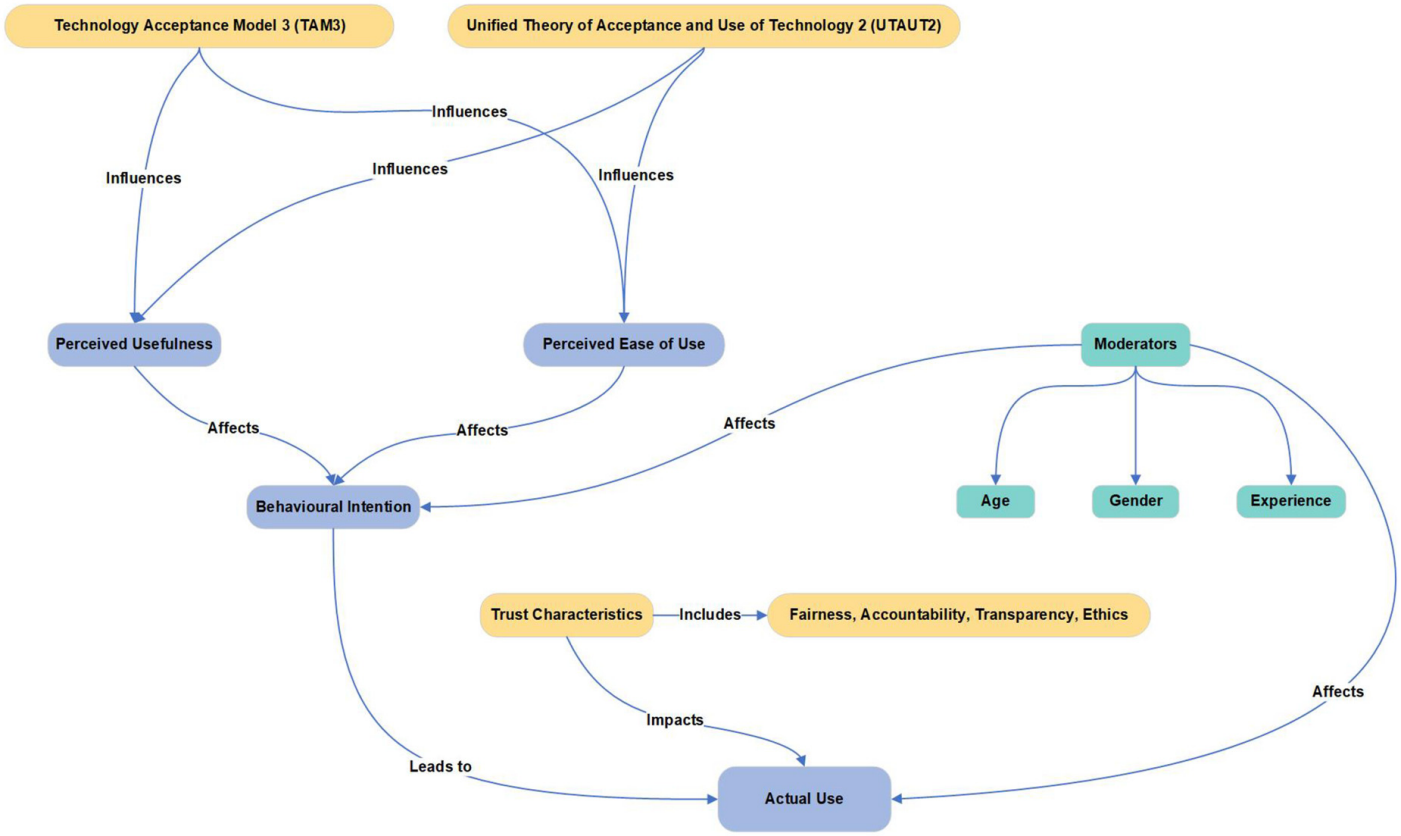
Conceptual framework combining technology acceptance model 3 (TAM3), unified theory of acceptance and use of technology 2 (UTAUT2) and trust characteristics.

Using the conceptual framework (Figure 1), the study formulated a series of hypotheses that structured the online survey into key areas: acceptance levels, stakeholder influence, barriers and benefits of AI in primary care. The survey questions were systematically developed through an iterative process by the authors with reference to validated instruments cited in previous studies^23,24^; no copyrighted material was directly reused, and all items were original formulations based on established constructs (Supplemental Material Appendix A). To ensure quality and rigour, three AI experts contributed to question development, and approval was obtained from the Open University Human Research Ethics Committee (HREC). The survey incorporated a mix of closed-ended, open-ended, Likert scale, rating and multiple-choice questions. This mixed-question format was designed to balance quantitative comparability with qualitative depth, enabling both statistical analysis and richer insights into stakeholder attitudes.

To enhance validity, the survey was pilot tested with five primary care employees not involved in the main study and three colleagues to assess clarity, flow and device readability. Pilot testing ensured that participants comprehended the questions as intended and provided expected responses.^25^ Based on feedback, revisions were made to question wording and sequencing.^18^ The online survey captured participants’ perceptions of AI without prior explanation, intentionally assessing their existing knowledge and understanding. To establish rigour and internal consistency, Cronbach's Alpha was calculated for each section of questions. All key constructs demonstrated acceptable reliability (α≥0.70).^26^ Exact alpha values can be found in Appendix A.

### Interview design

Semi-structured interviews were conducted with a purposively selected subset of survey respondents, who expressed interest in follow-up discussions (Appendix A). The interviews were conducted via secure video conferencing, recorded with consent, and automatically transcribed. At the start of each interview, AI was defined as: ‘The method by which a computer is able to act on data (text, speech or images) through statistical analysis, enabling it to understand, analyse, and learn from data through specifically designed algorithms’. Participants were then presented with four scenarios representing AI applications in classification, prediction, optimisation and generation (Appendix B). These scenarios drawn from various sectors, aimed to prompt participants to discuss AI applications relevant to their daily practice, reducing response bias. The automatic transcriptions were subsequently reviewed for accuracy, cleaned and pseudonymised. A denaturalised transcription approach was used, meaning non-verbal utterances and speech disfluencies were removed while preserving meaning, ensuring clarity in qualitative analysis. The denaturalised approach prioritised the content of responses, in contrast to the naturalised approach which focuses on how statements are articulated.^27^

### Reflexivity

The interviewer had previously worked within primary care in an administrative role. The participants were given consent forms and participant information sheets prior to the interviews. Peer debriefing supported reflexivity during data collection and interpretation, helping to identify and account for potential bias.

### Data analysis and validation

Quantitative survey data were analysed using descriptive statistics and inferential tests, including ANOVA to explore differences across stakeholder levels. However, this paper primarily reports on the qualitative findings derived from the open-ended survey questions and semi-structured interviews. The quantitative analyses are available in Appendix A but are not the focus of this manuscript.

**Figure 2.**
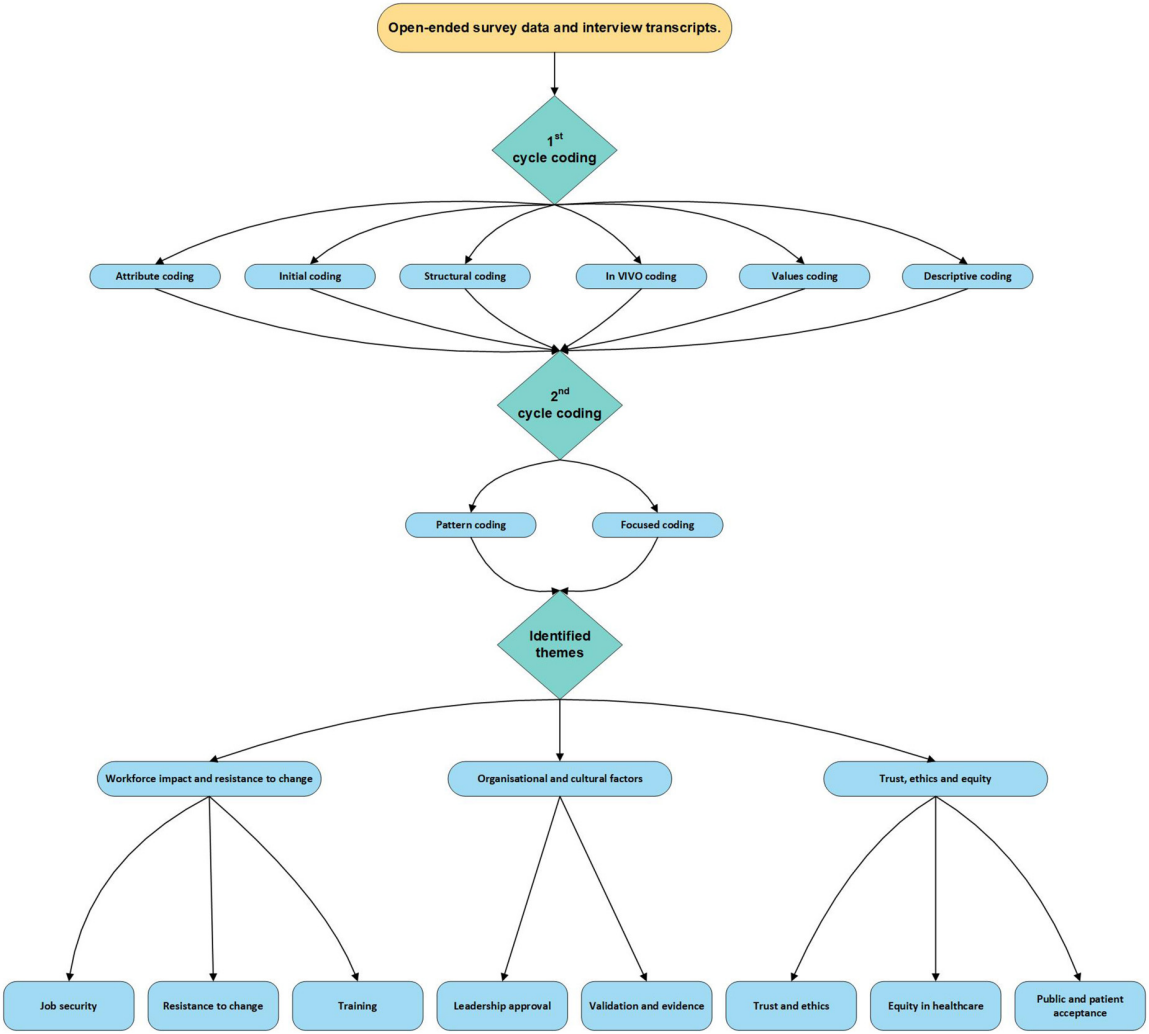
Flowchart of coding process.

Qualitative data were examined through an abductive thematic analysis approach, ensuring both data-driven and theory-informed insights.^28^ This method was selected to critically examine existing knowledge by integrating inductive and deductive codes with new observations, providing a more comprehensive understanding of the data.^29^ To ensure analytical depth and rigour, thematic coding employed multiple coding techniques – attribute, initial, structural, inVivo, values and descriptive^30^ – to identify key themes and patterns within the data (as illustrated in Figure 2).^28,30^ All qualitative data were managed and coded in NVivo. Coding was conducted iteratively, incorporating attribute coding to categorise participants by stakeholder level, gender and age enabling between-subjects analysis. The themes were refined through peer discussion with three AI experts. A visual representation of the coding process is shown in Figure 2. The adequacy of the qualitative sample was justified using the concept of information power.^31^ Specifically, the study's narrow focus, strong theoretical underpinning, and inclusion of informed participants with relevant primary care experience supported the sufficiency of the data collected. This approach aligned with the principles of reflexive thematic analysis.^32^

## Results

A total of 133 participants completed the online survey, and 9 participated in follow-up interviews. Interviewees represented both micro- and meso-level stakeholder roles. Unfortunately, no macro-level stakeholders opted into the interviews, representing a limitation in the diversity of qualitative responses. Table 1 outlines the demographic and stakeholder level composition of both samples.

**Table 1.** Demographic counts and percentages for survey and interviews.

	Online survey n (%)	Interview n (%)
Population	133 (100%)	9 (100%)
Macro-level	31 (23.3%)	0
Meso-level	49 (36.8%)	3 (33.3%)
Micro-level	53 (39.8%)	6 (66.7%)
Female	98 (73.7%)	7 (77.8%)
Male	31 (23.3%)	2 (22.2%)
Non-binary	2 (1.5%)	0
Prefer not to say	2 (1.5%)	0

Three key themes were identified through analysis of open-ended survey responses and interview transcripts (as shown in Figure 2).

### Workforce impact and resistance to change

**Figure 3.**
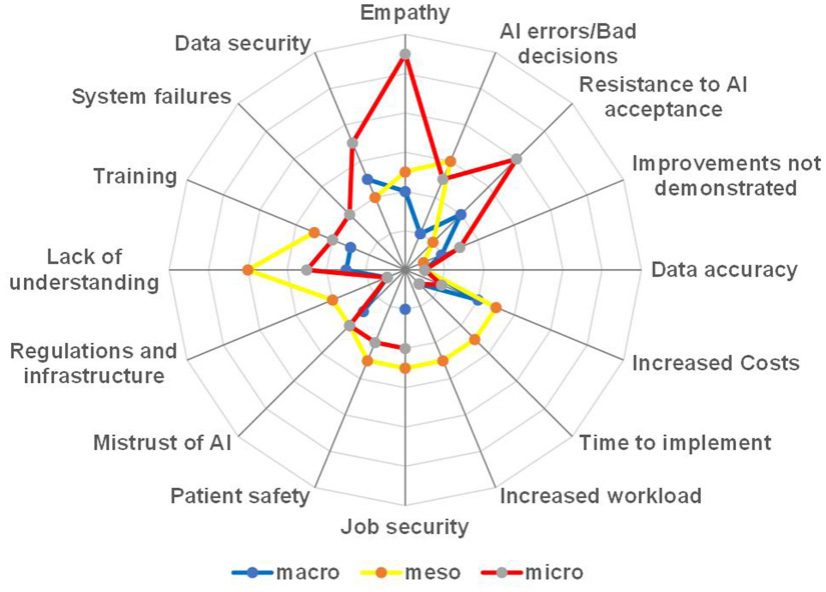
Barriers identified in online survey open-ended questions.

This theme captured concerns regarding AI's effect on employment, skills gaps and resistance to technological adoption. Approximately 13% (15 out of 118) of survey respondents (Figure 3) and a significant majority, 78% (n = 7) of interview participants, cited job security concerns as a key barrier to AI acceptance.

One participant noted:

‘People losing their roles and people having to take a bit of a back seat to computers’

(P42, meso, interview).

Additionally, another described the politicisation of job loss concerns:

‘I think it could be quite interesting from a political point of view and staffing point of view and unions being worried about people losing their jobs’

(P33, micro, interview).

Further, a participant described how colleagues reacted with anxiety when learning about his automated visual assessment project:

‘When I’ve been discussing the project, I’m working on, which is automated visual assessment, the first thing they would say was, Why are you building your own replacement?’

(P56, micro, interview).

Whereas a macro-level participant (P76, macro, survey) specifically identified the fact that introducing AI would reduce staff: ‘*Reduction of job roles for current staff’.*

### Organisational and cultural barriers

This theme addressed the influence of leadership approval, validation and integration into healthcare settings. Meso-level stakeholders expressed concerns over insufficient evidence of reliability and effectiveness.

One participant reflected on the accountability challenges of using AI in clinical decisions, questioning how responsibility would be assigned if outcomes were poor:

‘AI should demonstrate that it can accurately make decisions but who would be responsible if it went wrong? How could we be sure it's always accurate’

(P132, meso, interview).

Another participant highlighted the risks of over-reliance on AI expressing concerns that staff could make incorrect decisions based on automated recommendations, particularly if the systems lack transparency or reliability:

‘Making wrong decisions based on AI processes’

(P95, macro, survey).

Others stressed the importance of demonstrable value, stating that AI needs to be clearly validated before it could be accepted in practice:

‘For AI to be accepted in my role I’d need to see evidence of how it could operate’

(P128, meso, survey).

Transparency in AI decision-making was also identified as a critical factor in fostering trust, particularly in healthcare:

‘It's important for AI to explain how it makes decisions. This transparency is crucial, especially in fields like healthcare, where people's lives are at stake’

(P55, micro, interview).

Together, these views reflect how concerns around reliability, responsibility, and transparency create organisational and cultural barriers to AI adoption. Without clear validation mechanisms and leadership support, participants feared that AI may disrupt rather than support established workflows.

### Trust, ethics and equity

This theme encompassed issues related to trust in AI systems, ethical considerations and the potential for AI to exacerbate healthcare disparities. Specifically, 78% of stakeholders worried AI could exacerbate healthcare disparities if not implemented equitably, this was barely touched upon in the survey data with only 3% of participants highlighting inequity as a concern. Findings indicate that while AI may improve healthcare efficiency, concerns persist about exacerbating existing disparities, particularly regarding access and digital literacy.

Participants highlighted the risk of AI disproportionately benefiting already advantaged populations, reinforcing the inverse care law:

‘With new technologies you end up facilitating things for people that have already got the best experience and care. So, it's just the inverse care law at work’

(P101, micro, interview).

Other participants anticipated regional disparities in AI adoption:

‘Patterns of service would change nationally, with places using AI against

those that don’t use it’

(P33, micro, interview).

Stakeholders also noted that disparities could emerge from varying levels of AI adoption due to trust or technological access issues:

‘Potentially there could be disparities, if certain people choose not to use AI, maybe don’t have the technology needed or lack confidence’

(P59, micro, interview).

To mitigate this risk, policymakers should prioritise AI deployment in underserved areas, ensuring equitable access to AI-driven healthcare innovations.

Additionally, 16% of survey respondents and 67% (n = 6) of interview participants highlighted AI's lack of empathy as a factor affecting trust. One participant noted: ‘*Decisions made need to be ethical. Human decisions need to draw upon emotion as well as logic and need to be informed by compassion. Connection to other humans is key - would an AI reduce that?’* (P34, micro, survey).

Additionally, P3 (macro, survey) was concerned with the reduction in human contact: ‘*AI would reduce face-to-face contact with patients’.*

Another participant raised concerns about accountability:

‘AI can’t be prosecuted or lose its registration to practice’

(P85, meso, survey).

This concern highlights a key accountability gap in AI governance, wherein responsibility for AI-driven decisions remains ambiguous. Addressing this issue requires clear legal frameworks ensuring human oversight in AI-assisted diagnostics and decision-making.

**Figure 4.**
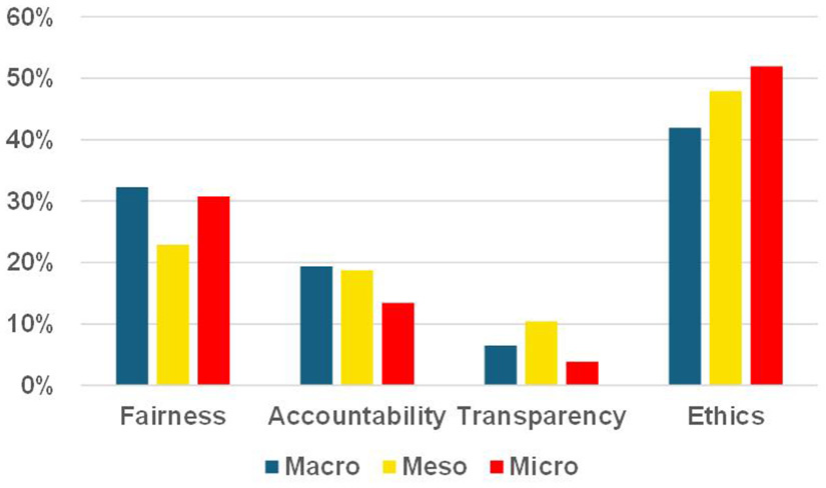
Perceived importance of trust characteristics.

When survey participants ranked trust characteristics, ethical considerations emerged as the most important across all stakeholder levels, followed by fairness, accountability and transparency (Figure 4). This consensus underscored the shared priorities among stakeholders. The UK government recognised the significance of ethical considerations in AI governance through its ‘Ethics, Transparency and Accountability Framework for Automated Decision-Making’.^33^ To assess statistical significance in trust characteristic rankings, Friedman's tests were conducted, followed by Wilcoxon signed rank tests for comparative analysis. Cohen's d effect size calculations further established the reliability of these findings (Table 2).

**Table 2.** Matrix of significance and effect size for trust.

	Variable	Fair	Accountable	Transparent
Accountable	***z***	−1.11		
	***p***	0.27		
	***d***	0.10		
Transparent	***z***	−3.58	−2.26	
	***p***	<0.001	0.23	
	***d***	0.31	0.20	
Ethical	***z***	−2.57	−3.83	−5.89
	***p***	0.01	<0.001	<0.001
	***d***	0.22	0.33	0.51

### Stakeholder influence on AI acceptance

**Figure 5.**
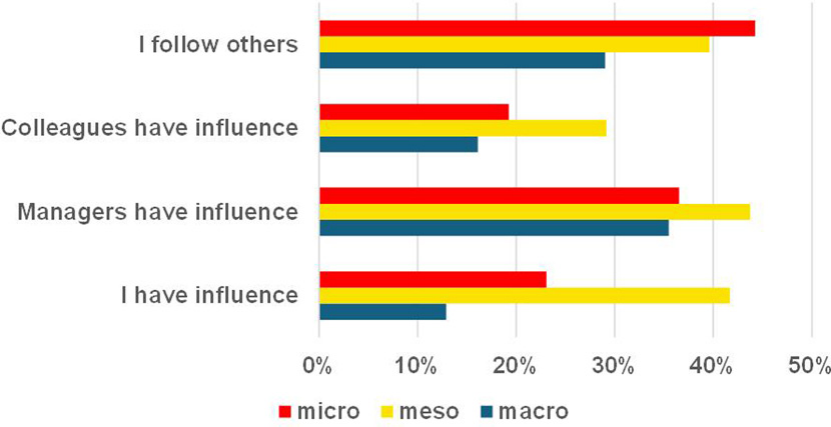
Perceived stakeholder influence over AI introduction.

An analysis of stakeholders perceived influence on AI implementation revealed significant variations, as illustrated in Figure 5. Meso-level stakeholders most frequently reported holding influence (N 20, 42%), whereas only 4 (13%) of macro-level stakeholders perceived themselves as influential. Furthermore, 21 (44%) of meso-level stakeholders believed macro-level stakeholders expected them to adopt AI, while 23 (44%) of micro-level stakeholders expressed willingness to use AI if others did so. These findings positioned meso-level stakeholders as pivotal to AI acceptance in primary care, underscoring the necessity of engaging with them for successful implementation. During interviews, meso-level stakeholders identified macro-level stakeholders as having authority over AI approval:

‘There needs to be senior leadership buy in, where people can explain the benefits of why AI is useful to primary care’

(P128, meso).

‘It [AI] would need to be approved by the NHS’

(P132, meso).

## Discussion

This study explored stakeholder perceptions of AI adoption in primary care, identifying key themes of workforce impact, organisational and cultural barriers, trust, ethics, equity and stakeholder influence. The findings align with existing literature, highlighting both challenges and facilitators of AI acceptance.

### Workforce impact and resistance to change

Stakeholders expressed concerns that AI implementation might lead to job losses, particularly if efficiency gains were prioritised over workforce retention. These concerns align with prior studies indicating that perceptions of AI as a cost-cutting tool can reduce stakeholder trust.^35^ However, this study add nuance by showing that such concerns are not confined to frontline staff but are also expressed at meso-level. This reflects Castagno and Khalifa's^11^ findings that healthcare professionals feel underinformed and vulnerable in the face of digital transformation.

As echoed in prior work,^36,37^ participants noted that AI lacks the relational capabilities – such as empathy and context-based reasoning – that define much of healthcare practice. This reinforces the need to frame AI as a supportive tool rather than a human replacement. Addressing these concerns requires clear policy guidelines, reskilling initiatives, and gradual AI integration to ensure workforce sustainability.

### Organisational and cultural barriers

AI acceptance in primary care is heavily influenced by organisational readiness, leadership approval, validation and evidence of reliability. In this study, meso-level stakeholders – such as practice managers and operational leads – consistently stressed the need for validation mechanisms before AI could be widely adopted. These findings echo broader concerns in the literature regarding the lack of regulatory frameworks and clinical validation in AI deployment.^38^ For instance, Castagno and Khalifa^11^ found that healthcare staff often express uncertainty around how AI is evaluated and regulated, which in turn erodes confidence in its use.

In contrast to integrated systems such as those in Canada – where national-level AI policy is more consistently operationalised – UK primary care is characterised by local variation in digital maturity and leadership readiness, which complicates AI integration at scale.^6^ This highlights the importance of engaging meso-level managers as local change agents within a decentralised NHS context.

Participants also raised concerns about the transparency of AI systems, particularly in the context of clinical decision-making. Here, transparency was framed operationally – can staff audit, understand and justify AI supported decisions? This aligns with Adadi and Berrada's^39^ work on explainable AI, which highlights that black-box models inhibit adoption unless their outputs are interpretable and clinically meaningful for end-users.

These findings underline that organisational or operational trust is distinct from ethical concerns and depends on practical mechanisms such as evidence-based implementation, clear accountability and regulatory alignment. A systematic review by Tun et al.^40^ identified system transparency, training and familiarity, usability and clinical reliability as critical drivers of clinician trust in AI-based systems.

### Trust, ethics and equity

Trust emerged as a central theme, particularly around ethical concerns, transparency and fairness. Ethical considerations were ranked as the most important factor across all stakeholder levels. This echoes findings from Starke et al.,^41^ who identified ethical alignment with system transparency as foundational to building trust in healthcare AI across international contexts. Accountability and transparency were also identified as critical trust factors, consistent with broader discussions on AI governance.^3^

Moreover, the study contributes to debates on AI and health equity. Concerns that AI could exacerbate existing inequalities were prominent in interviews, particularly in relation to the inverse care law – the principle that those who most need care are least likely to receive it.^42^ This reflects recent findings by Green et al.^43^, who highlighted concerns that AI could amplify existing healthcare disparities if social determinants, digital divides and bias in training data are not adequately addressed. Participants in this study also anticipated regional variations in AI adoption, suggesting that disparities may arise from differential access to AI technologies and varying levels of digital literacy.^44^

To mitigate these risks, future implementations must prioritise equitable deployment and include vulnerable populations in both design and rollout. Ethical AI adoption in primary care must account for differences in digital literacy, infrastructure and socio-economic conditions – not just system performance.

### Stakeholder influence on AI acceptance

This study highlights the role of meso-level stakeholders – such as practice managers – in mediating AI adoption in primary care. These actors perceived themselves as both subject to top-down policy pressures and responsible for operationalising AI locally. This dual role is underrepresented in existing research, which has tended to focus on either frontline (micro-level) or policy (macro-level) perspectives.^5,7,10^

These findings suggest that AI implementation efforts must actively engage meso-level stakeholders by equipping them with decision-support tools, AI literacy training and platforms for feedback. Their position between strategic directives and frontline realities makes them essential for translating policy into practice.

## Limitations

This study employed an online survey and semi-structured interviews to explore stakeholder perspectives on AI in NHS primary care. While the mixed-methods design provided depth and breadth, several limitations must be acknowledged.

The survey was distributed via Prolific, which facilitated access to a large pool of healthcare stakeholders but did not allow for tracking response rates, limiting recruitment transparency. Although stratification was applied via a pre-survey, to target stakeholder levels (macro, meso and micro), demographic quotas were not enforced, and participant representativeness relative to broader NHS primary care workforce cannot be guaranteed. Online-only data collection may have excluded individuals with limited digital literacy or access, potentially affecting the inclusivity of the sample.

Self-selection and snowball sampling may have introduced bias, particularly favouring individuals with a pre-existing interest in AI or technology. Although financial incentives were offered for participation, the low interview uptake – only 9 out of 72 interested respondents (12.5%) – suggests these did not significantly influence engagement, in line with prior findings.^34^

A further limitation is the absence of macro-level stakeholders in the interviews, restricting insight into strategic and policy-level decision-making regarding AI. While both meso- and micro-level perspectives were included, this omission limits the comprehensiveness of the qualitative findings across the organisational hierarchy.

Although the qualitative sample size was small (n = 9), saturation was reached when not new codes or themes emerged across three consecutive interviews. This aligns with the principle of information power, whereby a smaller, focused, and theoretically informed sample can still yield valid insights.^31^

## Conclusions

This study highlights the complexities of AI adoption in primary care, demonstrating that workforce concerns, organisational barriers, trust, ethics and stakeholder influence all play critical roles. The findings support prior research while also offering new insights into the contextual factors shaping AI acceptance. Addressing these challenges requires strategic oversight, inclusive stakeholder representation and a commitment to equitable implementation. To enhance trust in AI technologies, targeted interventions need to focus on positioning AI as a workforce-supporting tool. Additionally, establishing clear validation mechanisms and accountability structures, and fostering trust with ethical AI governance are essential. These efforts should also ensure equitable access to AI technologies and empower meso-level stakeholders through training and decisions-support initiatives. Future research should conduct longitudinal studies assessing AI adoption trends across different NHS regions, with a focus on evaluating whether stakeholder engagement strategies improve long-term trust and equity.

## Supplemental Material

sj-xlsx-1-dhj-10.1177_20552076251386706 - Supplemental material for Bridging trust gaps: Stakeholder perspectives on AI adoption in the United Kingdom NHS primary care Supplemental material, sj-xlsx-1-dhj-10.1177_20552076251386706 for Bridging trust gaps: Stakeholder perspectives on AI adoption in the United Kingdom NHS primary care by Teresa Sides, Dhouha Kbaier, Tracie Farrell and Aisling Third in DIGITAL HEALTH

sj-docx-2-dhj-10.1177_20552076251386706 - Supplemental material for Bridging trust gaps: Stakeholder perspectives on AI adoption in the United Kingdom NHS primary care Supplemental material, sj-docx-2-dhj-10.1177_20552076251386706 for Bridging trust gaps: Stakeholder perspectives on AI adoption in the United Kingdom NHS primary care by Teresa Sides, Dhouha Kbaier, Tracie Farrell and Aisling Third in DIGITAL HEALTH

sj-docx-3-dhj-10.1177_20552076251386706 - Supplemental material for Bridging trust gaps: Stakeholder perspectives on AI adoption in the United Kingdom NHS primary care Supplemental material, sj-docx-3-dhj-10.1177_20552076251386706 for Bridging trust gaps: Stakeholder perspectives on AI adoption in the United Kingdom NHS primary care by Teresa Sides, Dhouha Kbaier, Tracie Farrell and Aisling Third in DIGITAL HEALTH
