# Supplementary material for: Bridging trust gaps: Stakeholder perspectives on AI adoption in the United Kingdom NHS primary care
Source: Digit Health. 2025 Nov 27;11:20552076251386706. doi: 10.1177/20552076251386706 (PMC12660648; doi:10.1177/20552076251386706)
Supplement: sj-docx-2-dhj-10.1177_20552076251386706 - Supplemental material for Bridging trust gaps: Stakeholder perspectives on AI adoption in the United Kingdom NHS primary care [file sj-docx-2-dhj-10.1177_20552076251386706.docx]

Interview Script

Thank you for participating in this interview. I am sure this is a busy time for everyone. I know I am looking forward to a hike this Saturday! What will you do to unwind over the weekend?

Today we’ll be talking about artificial intelligence (AI), what is your understanding of AI?

**Artificial intelligence is the method by which a computer is able to act on data (text, speech, images) through statistical analysis, enabling it to understand, analyse, and learn from data through specifically designed algorithms.**

Some areas where you may have used AI in your general life are: digital assistants. (Alexa, Siri) where algorithms are used to answer, “Frequently Asked Questions”, take or track orders and direct calls to relevant departments.

AI is used with maps to enable the GPS to identify areas of high traffic and offer alternative routes.

Text editors and autocorrects such as Wordtune, Grammarly and ChatGPT

1. What thoughts, feelings, and perspectives come to mind first when you think about AI?
2. How would you describe AI?
3. What is your understanding of AI?

I am now going to describe four scenarios of AI systems currently being used within other sectors of society.

Please consider how these systems could help in your job role?

In this first scenario let’s discuss an AI based identity verification system for an international airports check-in process. The passengers’ documents are scanned at passport control/check-in and the scanned images are then checked for watermarks or holograms to enable authentication. The system is able to establish authentications for all types of identity documents across the globe. Then an image of the passenger’s face is checked against the photographic ID.

Can you please give your first impression of this AI system?

Would you find any part of this AI system useful in your job role?

Do you need to authenticate documents in primary care?

Do you need to compare images in primary care?

Of all the suggestions / ideas generated by this scenario, which do you feel would have the most impact?

We all know someone who uses public transport or have used public transport ourselves. This system uses AI to determine passenger demand at different times of the day and across different days of the week. Using both real-time data and historic data demand can be assessed for local and national events, or sporting events such as football matches.

Can you please give your first impression of this AI system?

Would you find this AI system useful in your job role?

Does demand for services change within primary care? When? Where?

Of all the suggestions / ideas generated by this scenario, which do you feel would have the most impact?

This AI system used for tax and accounting brings a range of advantages especially for SME’s, such as automation of mundane tasks, personalised advice and real-time insights. Invoices and receipts are automatically categorised and managed in real-time (with updates for tax regulation changes), allowing for strategies to be tailored to the individual business. Anomalies are automatically highlighted reducing the time spent on audits and investigations.

Can you please give your first impression of this AI system?

Would you find this AI system useful in your job role?

What type of tasks could be automated within primary care?

Do you have specific regulations that need to be followed?

Of all the suggestions / ideas generated by this scenario, which do you feel would have the most impact?

We have all wasted time waiting in a queue (physical or on the phone) trying to arrange an appointment, This system uses chatbots/ digital assistants to enable customers to book appointments or ask questions without human intervention. The system learns from your interactions such as which day you always book an appointment for or the time of day your appointments usually are to personalise the options available to you. Automated text messages or emails also gather feedback from the customers to enable improvements.

Can you please give your first impression of this AI system?

Would you find this AI system useful in your job role?

Of all the suggestions / ideas generated by this scenario, which do you feel would have the most impact?

What would you want AI to do for primary care if there was no limit on its capabilities?

If there was no budget, no limitations and no suggestion is a bad suggestion.

Is there anything we didn’t touch on that you feel is important?
