# Supplementary material for: Bridging trust gaps: Stakeholder perspectives on AI adoption in the United Kingdom NHS primary care
Source: Digit Health. 2025 Nov 27;11:20552076251386706. doi: 10.1177/20552076251386706 (PMC12660648; doi:10.1177/20552076251386706)
Supplement: sj-docx-3-dhj-10.1177_20552076251386706 - Supplemental material for Bridging trust gaps: Stakeholder perspectives on AI adoption in the United Kingdom NHS primary care [file sj-docx-3-dhj-10.1177_20552076251386706.docx]

**Consolidated criteria for reporting qualitative studies (COREQ): 32-item checklist**

| **Item** | **Guide questions/description** | **Response** |
| --- | --- | --- |
| **Domain 1: Research team and reﬂexivity** | | |
| **Personal Characteristics** |  |  |
| 1. Interviewer/facilitator | Which author/s conducted the interview or focus group? | Teresa Sides (lead researcher) |
| 2. Credentials | What were the researcher’s credentials? E.g. PhD, MD | PhD student |
| 3. Occupation | What was their occupation at the time of the study? | Consultant researcher |
| 4. Gender | Was the researcher male or female? | Female |
| 5. Experience and training | What experience or training did the researcher have? | Formal training in thematic analysis |
| **Relationship with participants** |  |  |
| 6. Relationship established | Was a relationship established prior to study commencement? | Yes—participants had previously completed an online survey and indicated willingness to participate in further research. However, no personal relationship was established prior to the interviews. |
| 7. Participant knowledge of the interviewer | What did the participants know about the researcher? e.g. personal goals, reasons for doing the research | Participants received a participant information sheet and consent form prior to the interview. These documents outlined the research aims, the role of the interviewer, and what participation would involve. |
| 8. Interviewer characteristics | What characteristics were reported about the inter viewer/facilitator? e.g. Bias, assumptions, reasons and interests in the research topic | The interviewer had prior professional experience working in primary care, which informed the choice of research focus. Reflexivity was maintained throughout to acknowledge and manage potential bias arising from this background. |

| **Domain 2: study design** | | |
| --- | --- | --- |
| **Theoretical framework** |  |  |
| 9. Methodological orientation and Theory | What methodological orientation was stated to underpin the study? e.g. grounded theory, discourse analysis, ethnography, phenomenology, content analysis | Thematic analysis was used to analyse the data, following Braun and Clarke’s six-phase approach. |
| **Participant selection** |  |  |
| 10. Sampling | How were participants selected? e.g. purposive, convenience, consecutive, snowball | Participants were recruited from a previous online survey and invited email to take part in interviews. |
| 11. Method of approach | How were participants approached? e.g. face-to-face, telephone, mail, email | Participants were contacted via email using the contact details they provided in the initial survey. |
| 12. Sample size | How many participants were in the study? | Nine participants took part in the interviews |
| 13. Non-participation | How many people refused to participate or dropped out? Reasons? | Seventy participants were invited via email. Sixty-one did not respond or declined to participate, with no specific reasons provided. |
| *Setting* |  |  |
| 14. Setting of data collection | Where was the data collected? e.g. home, clinic, workplace | The interviews were conducted online via Microsoft Teams. Participants were given the option to keep their cameras off; the interviewer’s camera remained on throughout. |
| 15. Presence of non-participants | Was anyone else present besides the participants and researchers? | No non-participants were present during data collection. |
| 16. Description of sample | What are the important characteristics of the sample? e.g. demographic data, date | Participants were aged between 18 and 45, with mixed genders. Eight were based in England and one in Scotland. None were from Northern Ireland or Wales. All worked within primary care and had recent experience delivering services within that setting. |
| **Data collection** |  |  |
| 17. Interview guide | Were questions, prompts, guides provided by the authors? Was it pilot tested? | A semi-structured interview guide was used. It was pilot tested with three primary care employees who hadn’t taken part in the survey. A presentation was used to structure the interviews: it began by defining AI, followed by four scenarios illustrating AI systems in different societal sectors. These scenarios represented the four main forms of AI functionality—classification, prediction, optimisation and generation—to prompt discussion and reflection. |
| 18. Repeat interviews | Were repeat interviews carried out? If yes, how many? | No |
| 19. Audio/visual recording | Did the research use audio or visual recording to collect the data? | Microsoft Teams recorded the interviews and automatically transcribed them. |
| 20. Field notes | Were ﬁeld notes made during and/or after the interview or focus group? | Field notes were taken during and immediately after each session to capture contextual details and initial impressions. |
| 21. Duration | What was the duration of the interviews or focus group? | The interview sessions lasted between 45 and 70 minutes. |
| 22. Data saturation | Was data saturation discussed? | Data saturation was considered during analysis. Themes began to recur across participants, indicating saturation. |
| 23. Transcripts returned | Were transcripts returned to participants for comment and/or correction? | Transcripts were not returned to participants |
| **Domain 3: analysis and ﬁndings** |  |  |
| *Data analysis* |  |  |
| 24. Number of data coders | How many data coders coded the data? | One researcher conducted the initial coding. A second researcher reviewed a subset of transcripts to enhance reliability. |
| 25. Description of the coding tree | Did authors provide a description of the coding tree? | Yes. A codebook was developed and provided, containing all codes used in the analysis along with detailed descriptions for each code. |
| 26. Derivation of themes | Were themes identiﬁed in advance or derived from the data? | Themes were derived from the data using an abductive coding approach. First cycle coding included attribute, initial, structural, in vivo, values and descriptive coding. Second cycle coding involved pattern and focused coding to refine and consolidate themes. |
| 27. Software | What software, if applicable, was used to manage the data? | NVivo 15 was used to support thematic analysis, enabling efficient coding, organisation, and retrieval of qualitative data. |
| 28. Participant checking | Did participants provide feedback on the ﬁndings? | No. Participants were not involved in the analysis stage and did not provide feedback on the findings. |
| *Reporting* |  |  |
| 29. Quotations presented | Were participant quotations presented to illustrate the themes/ﬁndings? Was each quotation identiﬁed? e.g. participant number | Yes. Participant quotations were included to support each theme and were attributed using anonymized identifiers (e.g., P1, P2) |
| 30. Data and ﬁndings consistent | Was there consistency between the data presented and the ﬁndings? | Yes. The findings were grounded in the data, with clear connections between participant responses and the themes presented. |
| 31. Clarity of major themes | Were major themes clearly presented in the ﬁndings? | Yes. Major themes were clearly defined and supported by illustrative quotes. |
| 32. Clarity of minor themes | Is there a description of diverse cases or discussion of minor themes? | Yes. Minor themes and divergent views were acknowledged to reflect the range of participant experiences. |
